# 2′-Hydroxycinnamaldehyde ameliorates imiquimod-induced psoriasiform inflammation by targeting PKM2-STAT3 signaling in mice

**DOI:** 10.1038/s12276-021-00620-z

**Published:** 2021-05-14

**Authors:** Lihua Hao, Yuancheng Mao, Jin Park, Byoung-Mog Kwon, Eun Ju Bae, Byung-Hyun Park

**Affiliations:** 1 Department of Biochemistry and Molecular Biology, Chonbuk National University Medical School, Jeonju, Jeonbuk 54896 Republic of Korea; 2 Department of Dermatology, Chonbuk National University Medical School, Jeonju, Jeonbuk 54896 Republic of Korea; 3 Laboratory of Chemical Biology and Genomics, Korea Research Institute of Bioscience and Biotechnology, Daejeon, 34141 Republic of Korea; 4 College of Pharmacy, Chonbuk National University, Jeonju, Jeonbuk 54896 Republic of Korea

**Keywords:** Molecularly targeted therapy, Autoimmunity

## Abstract

2′-Hydroxycinnamaldehyde (HCA), the active component isolated from the stem bark of *Cinnamomum cassia*, exerts anticancer effects through multiple mechanisms. We recently determined that HCA inhibits signal transducer and activator of transcription 3 (STAT3) signaling in prostate cancer cells. Because STAT3 overactivation has been closely associated with the development of psoriasis, a chronic autoimmune skin disease, we examined whether HCA ameliorates skin lesions in an imiquimod-induced psoriasis-like mouse model. The results showed that intraperitoneal administration of HCA alleviated imiquimod-induced psoriasis-like dermatitis, epidermal thickening, dermal infiltration of inflammatory cells, and proinflammatory cytokine production. Mechanistically, HCA inhibited pyruvate kinase isozyme M2 and STAT3 signaling, leading to the suppression of T cell activation, Th17 cell differentiation, and keratinocyte hyperproliferation. These results suggest that HCA may be a new treatment for psoriasis and other STAT3-mediated skin disorders, such as infection, inflammation and carcinogenesis.

## Psoriasis: Cinnamon bark derivative reduces skin inflammation in mice

An active compound found in cinnamon bark could help alleviate the symptoms of psoriasis according to a study in mice by researchers in South Korea. A team led by Eun Ju Bae and Byung-Hyun Park from Chonbuk National University in Jeonju administered 2′-hydroxycinnamaldehyde, a molecule derived from cinnamon, to mice with drug-induced psoriatic skin lesions. After the treatment, the mice showed fewer signs of skin irritation, with less inflammation and no detectable ill effects. The researchers showed that the drug blocked an enzyme that normally activates a central regulator of immune responses in the skin. Consequently, fewer proinflammatory T cells infiltrated the skin and epidermal cells did not grow out of control. The findings highlight the potential of a natural product to safely treat psoriasis and related inflammatory disorders.

## Introduction

Psoriasis is a chronic autoimmune disease characterized by sharply demarcated, scaly, erythematous plaques often located on extensor surfaces^1^. Histological hallmarks of psoriatic skin lesions include hyperproliferation of keratinocytes, excessive dermal infiltration of inflammatory cells composed mainly of dendritic cells (DCs), T cells, macrophages and neutrophils, and increased dermal vascularity^2,3^. While the pathogenesis of psoriasis remains unclear, studies have revealed that the disruption of immune tolerance and concomitant excessive inflammatory cytokine production are initial events. Specifically, activated DCs produce several cytokines—notably interleukin (IL)-23 (IL-23), which activates and polarizes naïve T cells into Th17 cells, which are defined by the production of IL-17 and IL-22^4^. These cytokines interfere with the switch from keratinocyte proliferation to differentiation, ultimately leading to keratinocyte hyperproliferation and epidermal thickening. To support this event, increased levels of IL-23 and Th17 cytokines in serum and at skin lesions have been reported in psoriasis patients^5,6^, and biologics targeting these cytokines have been successful in improving psoriatic symptoms^7^, underscoring the importance of the IL-23-Th17 cytokine axis in psoriasis pathogenesis. At the molecular level, during the development of Th17 cells, IL-23 activates signal transducer and activator of transcription 3 (STAT3) to initiate gene transcription of RAR-related orphan receptor γt (RORγt), a master regulator in the development of Th17 cells^8,9^. Similarly, in keratinocytes, STAT3 is activated by Th17 cytokines IL-22 and IL-17A to stimulate cell proliferation and a feed-forward inflammatory response^10,11^. Thus, STAT3 activation is the central pathway for Th17 polarization and keratinocyte proliferation. In this context, several synthetic or natural STAT3 inhibitors have been shown to be highly effective in treating psoriatic symptoms in mice and humans^12–15^.

STAT3 transcriptional activity can be regulated by multiple activators and negative modulators. STAT3 is activated by phosphorylation at tyrosine (Y705) or serine (S727) residue in the transactivation domain, and the consequently formed STAT3 dimer translocates to the nucleus, where it promotes transcription of target genes. STAT3 phosphorylation at Y705 occurs primarily via Janus-activated kinase (JAK) family members, whereas phosphorylation at S727 is commonly performed by mitogen-activated protein kinases, cyclin-dependent kinase 5, and protein kinase C^16^. Canonical STAT3 signaling is further regulated by various feedback-inhibitory loops, including protein tyrosine phosphatases, suppressors of cytokine signaling (SOCS) proteins, and protein inhibitors of activated STAT (PIAS)^17,18^. We recently reported that pyruvate kinase isozyme M2 (PKM2) acts as an upstream kinase of STAT3 in imiquimod (IMQ)-induced psoriasiform skin lesions and that specific modulation of PKM2 activity is effective in improving disease severity^19^. Therefore, PKM2 inhibition and consequent STAT3 suppression seem to offer another therapeutic modality for psoriasis.

Both our group and others have reported that the cinnamaldehyde derivative 2′-hydroxycinnamaldehyde (HCA) exhibits anticancer effects by suppressing the STAT3 pathway and producing reactive oxygen species in a variety of in vitro and in vivo cancer models, including breast, head and neck, prostate, and oral cancer models^20–23^. In addition, we have ascertained that PKM2 is a direct target by which HCA suppresses STAT3 phosphorylation^24^. To date, however, the effect on psoriasis has yet to be reported. The present study was thus conducted to determine the therapeutic effect of HCA on psoriasiform skin lesions in C57BL/6 mice, as well as its underlying mechanisms.

## Materials and methods

### Animals

C57BL/6 mice were obtained from Orient Bio (Seoul, Korea). All animal experiments were performed in accordance with the Guide for the Care and Use of Laboratory Animals published by the US National Institutes of Health (NIH Publication No. 85-23, revised 2011). The current study protocol was approved by the Institutional Animal Care and Use Committee of Chonbuk National University (Approval no. CBNU 2020-0120).

### Cell culture and 5-bromo-2′-deoxyuridine (BrdU) assays

The human keratinocyte cell line HaCaT was obtained from the American Type Culture Collection (Manassas, VA, USA). HaCaT cells (3 × 10^3^) were seeded into a 96-well plate. Conditioned medium (CM) from Th17 cell cultures or IL-22 was added to the culture plate and incubated for 48 h, and cell proliferation was determined by a BrdU assay kit (Biovision, Milpitas, CA, USA).

### MTT assay for cell viability

Cell viability was determined by analyzing the reduction of 3-(4,5-dimethylthiazol-2-yl)-2,5-diphenyltetrazolium bromide (MTT) to formazan.

### CD4^+^ T cell preparation, T cell activation, and Th17 cell differentiation

Single-cell suspensions were prepared from spleens, and CD4^+^ T cells were isolated using a CD4^+^ T cell isolation kit (Miltenyi Biotec, San Diego, CA, USA). The purity of CD4^+^ T cell populations was ~95%, as assessed by Accuri C6 flow cytometry (BD Biosciences, San Jose, CA, USA). For T cell activation, purified CD4^+^ T cells were plated at a density of 1 × 10^6^ cells per well in wells that had been precoated with anti-CD3 antibodies (1 μg/ml). Anti-mouse CD28 antibodies (5 μg/ml) were added to the cell cultures. After 3 days, cell surface staining for CD25 and CD69 was performed. For Th17 cell differentiation, splenic CD4^+^ T cells (1 × 10^6^/ml) were seeded into a 24-well plate and polarized into Th17 cells by the addition of 1 μg/ml anti-CD3e antibodies, 5 μg/ml anti-CD28 antibodies, 10 μg/ml anti-IFN-γ antibodies, 10 μg/ml anti-IL-4 antibodies, 20 ng/ml IL-23, 20 ng/ml IL-6, 10 ng/ml TNF-α, and 2 ng/ml hTGFβ. All antibodies and recombinant cytokines were obtained from eBioscience (San Diego, CA, USA). Cell-free supernatant was collected after 3 days for cytokine detection by ELISA. Cells were collected after 5 days for flow cytometric and qPCR analyses.

### Psoriasiform skin inflammation models

To induce a mouse psoriasis model using IMQ, female mice at 9 weeks of age (20–22 g) were treated with a daily topical dose of 5% IMQ (62.5 mg Aldara cream, Dong-A ST, Seoul, Korea) for 6 consecutive days. Control mice were treated with the same volume of Vaseline (Unilever, London, UK). On day 7, the back skin was isolated for histopathological, gene expression, and flow cytometric analysis. The severity of skin inflammation was monitored and graded using a modified human Psoriasis Area Severity Index scoring system. Erythema, scaling, and thickening were scored independently from 0 to 4 (0 = none; 1 = slight; 2 = moderate; 3 = marked; and 4 = very marked). A cumulative score for each parameter served as a measure of inflammation severity (scale 0–12).

### Preparation of HCA

HCA was prepared as described previously^22^. Methotrexate (MTX) and HCA were dissolved in 4% DMSO + 6% Tween 80 + 10% dimethylacetamide + 80% PBS. Mice were injected intraperitoneally with HCA (10 or 30 mg/kg) or orally administered MTX (1 mg/kg) every day during the application of IMQ.

### Histology

For histopathology, the skin tissues were fixed in 10% formalin and embedded in paraffin. Section (5 μm) of back skin were stained with H&E to evaluate inflammation. After deparaffinization, the tissue sections were immunostained with antibodies against Ki67 (Novus Biologicals, Centennial, CO, USA).

### Flow cytometric analysis

Mouse skin tissues were isolated using collagenase IV enzymatic digestion. For surface staining, Fc receptors were blocked with mouse seroblockFcR (CD16/CD32, eBioscience). Cells were stained with FITC-conjugated anti-CD80, PE-conjugated anti-CD11c, FITC-conjugated anti-F4/80, PerCP-conjugated anti-CD11b, PE-conjugated anti-Ly6g, or APC-conjugated anti-CD11b antibodies for 30 min at 4 °C. To stain Th17, Th1, and γδT cells, cells were stimulated with a cell stimulation cocktail and Brefeldin A for 4 h. Cells were surface-stained with PerCP-conjugated CD4 or APC-conjugated CD3 with FITC-conjugated TCRγδ antibodies for 30 min on ice, fixed for 30 min, and washed twice with permeabilization buffer. Cells were stained using FITC-conjugated IL-17A, PE-conjugated IL-17A, or FITC-conjugated IFN-γ antibodies for 30 min at 4 °C in permeabilization buffer. After being washed with FACS buffer (3% FBS in PBS) three times, the cells were analyzed using an Accuri flow cytometer (BD Biosciences).

### Biochemical analysis

Serum levels of IL-22, IL-1β, IL-6, IL-17A, TNF-α, IL-10, and IL-23 were measured using ELISA kits (all from eBioscience). Serum levels of alanine aminotransferase (ALT) and aspartate aminotransferase (AST) were analyzed using specific kits (Asan Pharm, Seoul, Korea).

### Western blotting

Skin tissues were homogenized in tissue protein extraction reagent or mammalian protein extraction reagent (Thermo, Waltham, MA, USA). Tissue homogenates or cell lysates (20 μg of total protein) were separated by SDS-PAGE and transferred to nitrocellulose membranes. Blots were probed with primary antibodies against p-STAT1 (Y701), STAT1, p-STAT2 (Y690), STAT2, p-STAT3 (Y705), STAT3, p-STAT4 (Y693), STAT4, p-STAT5 (Y694), STAT5, p-STAT6 (Y641), STAT6, p-PKM2 (Y105), PKM2, Ac-lysine, p-JAK2 (Y1007/1008), JAK2, SOCS1, SOCS3, cyclin D1 (Cell Signaling Technology, Beverly, MA, USA), RORγt, c-myc (eBioscience), Sirt2, Lamin B (Santa Cruz Biotechnology, Dallas, TX, USA) and HSP90 (Enzo Life Sciences, Plymouth Meeting, PA, USA).

### PKM2 cross-linking assay

For the PKM2 cross-linking assay, cells were collected and washed three times with ice-cold PBS (pH = 8.0) to remove amine-containing culture media and proteins. Disuccinimidyl suberate solution was added to a final concentration of 1 μM and incubated for 30 min at room temperature. The cross-linking reaction was quenched by the addition of 10 mM Tris solution to the reaction mixtures. Finally, the cells were lysed with lysis buffer followed by Western blot analysis.

### RNA isolation and qPCR

Total RNA was extracted from cells or full-thickness back skin using TRIzol reagent (Invitrogen, Carlsbad, CA, USA). First-strand cDNA was generated by reverse transcription with oligo dT-adaptor primers (TaKaRa, Tokyo, Japan). Specific primers were designed using qPrimerDepot (http://mouseprimerdepot.nci.nih.gov, Table S1). The qPCR reactions had a final volume of 10 μl and contained 10 ng of reverse-transcribed total RNA, 200 nM forward and reverse primers, and PCR master mix. RT-PCR was performed in 384-well plates using an ABI Prism 7900HT Sequence Detection System (Applied Biosystems, Foster City, CA, USA).

### Statistical analysis

The data are expressed as the mean ± standard deviation (SD). Differences among groups were analyzed by a nonparametric Kruskal–Wallis test followed by Dunn’s multiple comparison test. A *p* value < 0.05 was considered significant.

## Results

### HCA attenuates IMQ-induced skin lesions

**Fig. 1:**
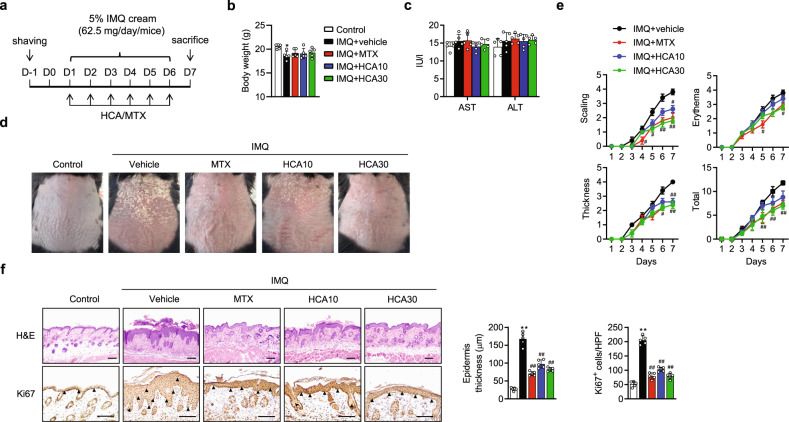
Attenuation of IMQ-induced psoriasiform skin symptoms by HCA. **a** Psoriasiform dermatitis was induced on the shaved back skin of C57BL/6 mice by topical IMQ application for 6 consecutive days, during which HCA (10 or 30 mg/kg) was administered intraperitoneally to the mice. MTX (1 mg/kg) was used as a positive control. **b, c** Body weight and serum levels of AST and ALT were measured. **d** Representative images of mouse back skin on day 7. **e** Daily mean disease severity as indicated by back skin scaling, erythema, and thickness in IMQ-treated mice (*n* = 5). **f** Representative H&E and Ki67 staining of back skin (bars = 200 µm). Epidermal thickness was measured in H&E-stained microphotographs, and the number of Ki67-positive cells in representative high-power fields (HPFs) was counted in three areas. Arrowhead, Ki67-positive cells. The values are the mean ± SD. *p < 0.05 and **p < 0.01 versus control; ^#^*p* < 0.05 and ^##^*p* < 0.01 versus IMQ + vehicle. IMQ imiquimod, MTX methotrexate, HCA10 2′-hydroxycinnamaldehyde 10 mg/kg, HCA30 2′-hydroxycinnamaldehyde 30 mg/kg.

To investigate whether HCA exerts antipsoriatic effects, we administered HCA intraperitoneally to a mouse model of psoriasis. Psoriasis was induced by topical IMQ application for 6 consecutive days (Fig. 1a). MTX was used as a positive control. HCA treatment at doses up to 30 mg/kg was not toxic to the mice, as body weight and serum levels of AST and ALT were not changed (Fig. 1b and c). On the 7th day, the IMQ-treated mice showed markedly thickened, erythematous, and scaly skin lesions compared with those of the control mice (Fig. 1d and e). In contrast, HCA-treated mice had smoother skin and less severe psoriasiform symptoms than untreated control mice. The improvement in skin inflammation by HCA treatment was further confirmed by histochemical analysis, which showed that epidermal thickness and the number of Ki67-positive keratinocytes were significantly lower in HCA-treated mice than in vehicle-treated mice (Fig. 1f).

**Fig. 2:**
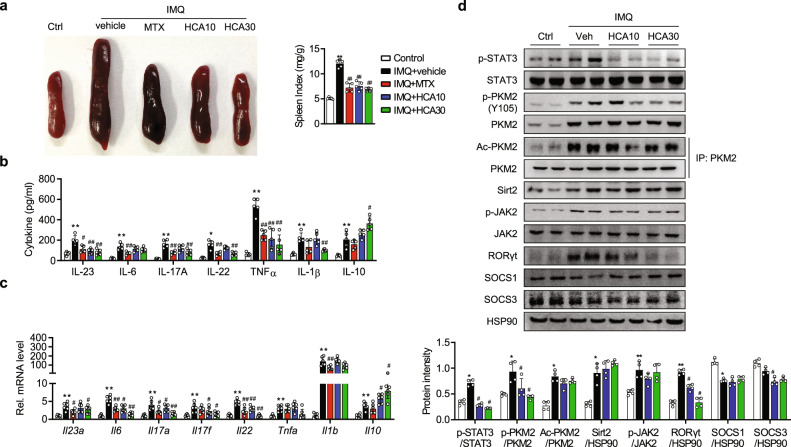
Attenuation of IMQ-induced psoriasiform skin inflammation by HCA. All experimental procedures are described in the legend of Fig. 1. **a** Representative photograph of spleens. The spleen index (spleen weight/body weight) was calculated. **b, c** Protein and mRNA levels of inflammatory cytokines in back skin were determined by ELISA and qPCR, respectively. **d** Protein levels of STAT3 and its positive and negative regulators in back skin were examined by Western blotting. The values are the mean ± SD. *p < 0.05 and **p < 0.01 versus control; ^#^*p* < 0.05 and ^##^*p* < 0.01 versus IMQ + vehicle. IMQ imiquimod, MTX methotrexate, HCA10 HCA 10 mg/kg, HCA30 HCA 30 mg/kg.

The spleen index was also diminished in the HCA group compared with the vehicle group, suggesting an anti-inflammatory effect of HCA (Fig. 2a). Consistently, the protein and mRNA levels of IL-23, IL-6, IL-17A, IL-22, and TNF-α were markedly reduced in HCA-treated mice (Fig. 2b and c). Our previous study showed that HCA inhibits the protein kinase activity of PKM2, which leads to the suppression of STAT3 phosphorylation in prostate cancer cells^24^. Accordingly, we examined whether this regulatory effect also occurred in psoriatic skin lesions. The results indicated that HCA treatment suppressed the phosphorylation of PKM2 and STAT3 (Fig. 2d). Other STAT family members, such as STAT2, STAT4, STAT5, and STAT6, were not affected by HCA treatment (Fig. S1). Importantly, the protein expression of RORγt was strongly induced by IMQ but markedly suppressed by HCA, which was consistent with p-STAT3 repression. Neither JAK2 phosphorylation nor SOCS1 and SOCS3 expression was affected by HCA (Fig. 2d), suggesting that HCA-mediated suppression of PKM2-STAT3 signaling is a key event in the attenuation of psoriasis development.

### HCA decreases the Th17 population in psoriasiform skin lesions

**Fig. 3:**
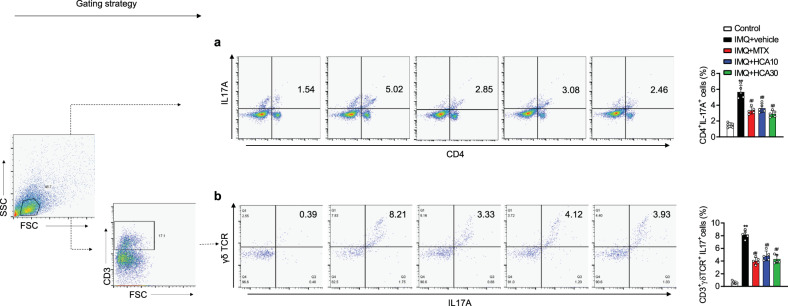
Flow cytometric analysis of back skin tissues. Cell suspensions were prepared by enzymatic digestion and gentle dissociation of back skin tissue and analyzed by flow cytometry. **a, b** Representative flow plots and subpopulations of Th17 cells (CD4^+^IL17A^+^) and IL-17-producing γδ T cells (CD3^+^γδ^+^IL17A^+^) are shown. The values are the mean ± SD. **p < 0.01 versus control; ^##^*p* < 0.01 versus IMQ + vehicle. HCA,10 HCA 10 mg/kg; HCA30 HCA 30 mg/kg.

Flow cytometric analysis of the lesional skin tissues of HCA-treated mice showed decreases in the numbers of Th17 cells (Fig. 3a), IL-17-producing γδ T cells (Fig. 3b), macrophages (Fig. S2a), DCs (Fig. S2b), and Th1 cells (Fig. S2c), while the number of neutrophils remained unchanged (Fig. S2d).

### HCA suppresses CD4^+^ T cell activation in vitro

**Fig. 4:**
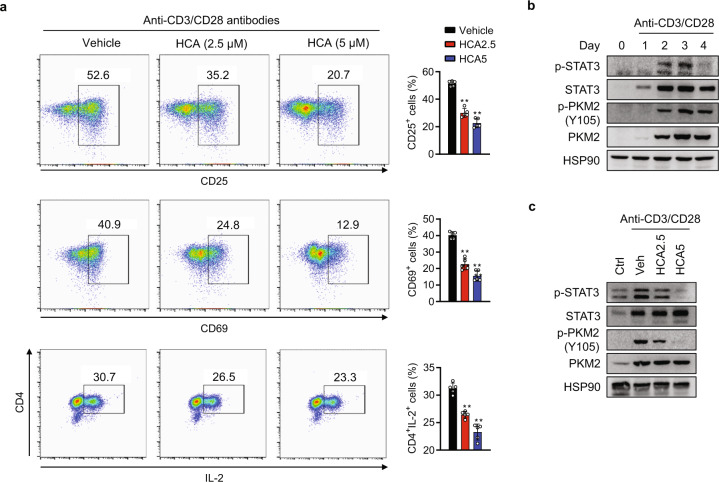
Effects of HCA on CD4^+^ T cell activation in vitro. Splenic naïve CD4^+^ T cells were stimulated with plate-bound anti-CD3 and soluble anti-CD28 antibodies and simultaneously treated with the indicated concentrations of HCA. **a** Surface expression of CD25 and CD69 and the number of CD4^+^IL-2^+^ T cells were determined by flow cytometry. **b** After CD4^+^ T cells were stimulated with anti-CD3/CD28 antibodies for the indicated times, the PKM2-STAT3 pathway was analyzed by Western blotting. **c** CD4^+^ T cells were stimulated with anti-CD3/CD28 antibodies for 3 days with or without the indicated concentrations of HCA, and the PKM2-STAT3 pathway was analyzed by Western blotting. The values are the mean ± SD. **p < 0.01 versus vehicle. HCA2.5 HCA 2.5 μM, HCA5 HCA 5 μM.

We next investigated whether HCA had an inhibitory effect on the activation of CD4^+^ T cells. Splenic CD4^+^ T cells were stimulated in vitro using anti-CD3/CD28 antibodies with or without HCA, and an assay was conducted to analyze surface markers of T cell activation, such as CD25 (IL-2Rα) and CD69, using flow cytometry. We first confirmed that HCA at concentrations up to 5 μM did not alter cell viability (Fig. S3a). The expression of CD25 and CD69 was significantly reduced by HCA treatment (Fig. 4a). This reduction was accompanied by significant suppression of the IL-2-expressing CD4^+^ T cell population in comparison with that of the untreated controls (Fig. 4a). Since suppression of PKM2 and STAT3 signaling by HCA was observed in psoriatic skin lesions, we investigated whether HCA could suppress PKM2-mediated STAT3 activation induced by anti-CD3/CD28 antibodies in CD4^+^ T cells. As shown in Fig. 4b and c, PKM2-STAT3 signaling was activated by anti-CD3/CD28 antibodies and markedly suppressed by HCA treatment, suggesting that HCA inhibits T cell activation by suppressing the PKM2-STAT3 pathway.

### HCA inhibits Th17 cell differentiation

**Fig. 5:**
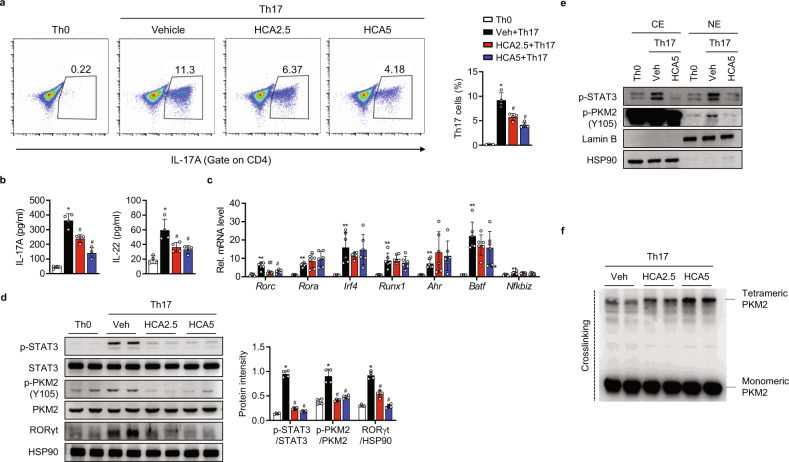
Suppression of Th17 cell differentiation by HCA. **a** Splenic naïve CD4^+^ T cells were cultured under neutral (Th0) or Th17-polarizing conditions. Subpopulations of Th17 cells were analyzed by flow cytometry. **b** IL-17A and IL-22 concentrations in culture supernatants on day 3 were analyzed by ELISA. **c** mRNA levels of transcription factors linked to Th17 cell differentiation were measured. **d** PKM2/STAT3 phosphorylation and RORγt expression on day 5 were analyzed by Western blotting. **e** Nuclear translocation of p-PKM2 on day 5 was analyzed by Western blotting. Lamin B was used as a loading control for nuclear proteins. **f** Western blot analysis of monomeric and tetrameric PKM2 in Th17 cells treated with HCA for 24 h. The values are the mean ± SD. *p < 0.05 and **p < 0.01 versus Th0; ^#^*p* < 0.05 and ^##^*p* < 0.01 versus Th17 + Veh. CE cytoslic extract, NE nuclear extract, HCA2.5 HCA 2.5 μM, HCA5 HCA 5 μM.

Having demonstrated the decreased production of Th17 cytokines in HCA-treated mice (Fig. 2b and c), we next investigated whether HCA impacted Th17 differentiation. Splenic CD4^+^ T cells were stimulated with anti-CD3 and anti-CD28 antibodies in the presence of a Th17-skewing cytokine cocktail. Intracellular staining followed by flow cytometric analysis revealed that HCA reduced the number of IL-17A^+^ cells within the CD4^+^ T cell population (Fig. 5a). This reduction was accompanied by impairment of IL-17A and IL-22 secretion in CD4^+^ T cells skewed toward the Th17 phenotype (Fig. 5b), indicating that HCA inhibits Th17 cell differentiation.

Among the various transcription factors linked to Th17 cell differentiation, the mRNA and protein levels of RORγt (*Rorc*) were selectively suppressed by HCA (Fig. 5c and d). Because RORγt is a downstream effector of STAT3 in CD4^+^ T cells, we next investigated the effects of HCA on PKM2-STAT3 signaling in CD4^+^ T cells. Consistent with the results observed in psoriatic skin tissues (Fig. 2d), HCA decreased the phosphorylation and nuclear translocation of PKM2 and subsequent STAT3 phosphorylation in CD4^+^ T cells (Fig. 5d and e). Moreover, a chemical cross-linking assay revealed that HCA promoted the association of PKM2 subunits as tetramers, which is known to suppress STAT3 phosphorylation (Fig. 5f). Taken together, these results suggest that HCA suppresses Th17 cell differentiation by inhibiting PKM2-STAT3 signaling and downregulating RORγt expression.

### HCA attenuates the IL-22 response in keratinocytes

**Fig. 6:**
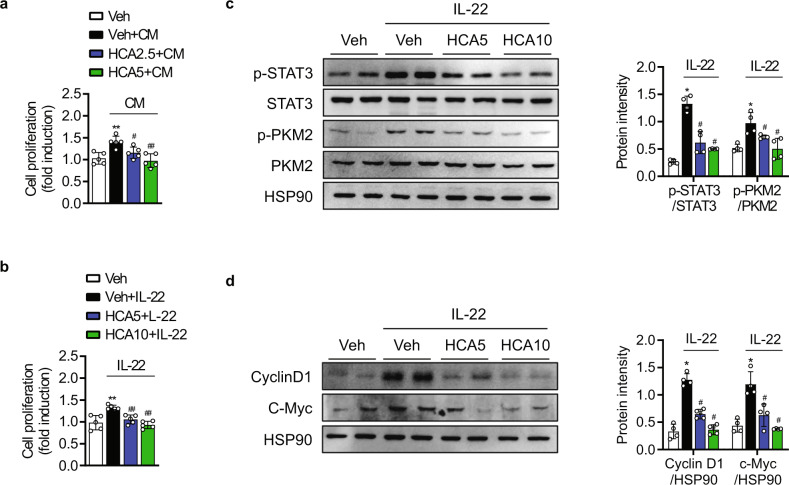
Suppression of HaCaT cell proliferation by HCA. **a, b** HaCaT cells were treated with conditioned medium (CM) from HCA-treated Th17 cell cultures or IL-22 (50 ng/ml) for 48 h, and then cell proliferation rates were measured by BrdU assays. **c** HaCaT cells were treated with IL-22 (50 ng/ml) for 30 min, and the JAK2/STAT3 pathway was analyzed by Western blotting. **d** HaCaT cells were treated with IL-22 (50 ng/ml) for 24 h, and the protein levels of cyclin D1 and c-Myc were analyzed by Western blotting. The value are the mean ± SD. *p < 0.05 and **p < 0.01 versus Veh; ^#^*p* < 0.05 and ^##^*p* < 0.01 versus Veh+CM or Veh+IL-22. HCA2.5 HCA 2.5 μM, HCA5 HCA 5 μM, HCA10 HCA 10 μM.

In view of the beneficial inhibitory effect of HCA on CD4^+^ T cells, we next investigated the impact of HCA-induced Th17 pathway inhibition on the keratinocyte response. We collected CM from Th17 cells that were incubated with or without HCA for 5 days. After activating HaCaT cells with Th17-derived CM for 48 h without HCA treatment, there was an increase in the cell proliferation rate (Fig. 6a). In contrast, CM obtained from Th17 cells in the presence of HCA induced a significant reduction in the cell proliferation rate.

Alternatively, we directly added recombinant IL-22 to HaCaT cells. Similarly, IL-22 treatment increased the cell proliferation rate (Fig. 6b), activation of the PKM2-STAT3 pathway (Fig. 6c), and the expression of c-Myc and cyclin D1 (Fig. 6d), suggesting that IL-22 is one of the main cytokines present in Th17 CM that activates HaCaT cells. However, HCA treatment significantly suppressed IL-22-stimulated HaCaT cell proliferation, PKM2-STAT3 activation, and c-Myc and cyclin D1 expression. HCA at the concentrations used did not affect the viability of HaCaT cells (Fig. S3b).

## Discussion

In this study, using IMQ-induced psoriatic mouse models, we were able to demonstrate the antipsoriatic effects of HCA. The antipsoriatic effect of HCA on mice was primarily attributable to its suppressive effect on STAT3 phosphorylation. Further in vitro analysis revealed that HCA treatment suppressed STAT3 activation in T cells, which is an initial event in the differentiation of naïve T cells into IL-22-secreting Th17 cells, as well as in the consequent proliferation of keratinocytes upon IL-22 receptor activation. It is well known that IMQ is a ligand of Toll-like receptor 7 (and TLR8 in humans) and recapitulates some of the immunological events in psoriasis patients^25^. Taking our current results and published reports into consideration, we propose the following sequence of events. IMQ treatment induces the secretion of IL-23 by DCs, which is essential for the differentiation of Th17 cells. These cells secrete the Th17 cytokines IL-17 and IL-22. IL-22 drives hyperproliferation and incomplete keratinocyte differentiation, leading to the formation of psoriatic plaques. In this context, HCA-mediated suppression of STAT3 in both Th17 cells and keratinocytes blocks the cellular event that triggers disease onset.

The HCA-mediated decrease in the spleen index suggests its systemic immunomodulatory effects. Because Th17 cells are a key player in the pathogenesis of psoriasis development^1,26^, we focused on the impact of HCA on Th17 cell function. Based on our previous finding that HCA binds directly to PKM2 to decrease STAT3 phosphorylation in prostate cancer cells^24^ and a study by Harris et al. showing that RORγt is a downstream effector of the STAT3 pathway and is linked to Th17 differentiation^27^, we investigated whether HCA affected the PKM2-STAT3 pathway in Th17 cells. It was evident that HCA treatment decreased the population of Th17 cells and concomitant Th17 cytokine production in psoriasiform skin lesions. Consistent with this finding, HCA also repressed RORγt expression, resulting in decreased CD4^+^ T cell differentiation into Th17 cells. We then further analyzed several positive and negative regulators of STAT3 activation. Among those examined, PKM2 phosphorylation was selectively suppressed by HCA. These results indicate that HCA suppression of PKM2 phosphorylation interferes with STAT3 signaling, thus inhibiting Th17 cell differentiation. Similar to our current findings, Angiari et al. reported that TEPP-46, a small molecule inhibitor of PKM2 nuclear translocation, suppressed Th17 cell differentiation and ameliorated experimental autoimmune encephalomyelitis, although direct evidence of STAT3 suppression was not provided^28^. Consistent with that study, our results suggest that HCA-mediated suppression of the PKM2-STAT3 axis may offer a useful therapeutic strategy for the treatment of psoriasis and Th17 cell-related autoimmune diseases.

Recent studies have highlighted the novel role of IL-17-producing dermal γδ T cells in psoriasis. While Th17 cells play a major role in the human form of psoriasis, γδT cells are a major source of IL-17 in IMQ-induced psoriasiform skin lesions^29^. Flow cytometric analysis confirmed that the application of IMQ demonstrably increased the proportion and absolute number of dermal γδT cells and confirmed that this effect was inhibited when HCA was administered. This finding suggests that HCA has therapeutic potential as a psoriasis treatment via the inhibition of γδ T cell function. However, because γδ T cells migrate to, proliferate, and differentiate in the draining lymph nodes, whereupon they are recruited to psoriatic lesions to amplify inflammation, further study will be required to determine whether HCA inhibits their recruitment or migration^30^.

We next observed a significant decrease in the number of Ki67-positive cells in psoriasiform skin lesions in response to HCA treatment, indicating that keratinocyte proliferation was also efficiently suppressed. An in vitro study using HaCaT cells further showed that HCA suppressed IL-22-induced keratinocyte proliferation. In parallel with current research, our previous studies confirmed the reduced proliferation of prostate cancer cells in the presence of HCA, which was attributable to decreased STAT3 phosphorylation^22,24^. Although the molecular mechanisms of psoriasis and cancer pathogenesis are quite different, both diseases are commonly characterized by hyperproliferation and abnormal differentiation of cells. Given that STAT3 signaling is activated by a variety of growth factors and cytokines and that STAT3 activation is instrumental in modulating cell proliferation and differentiation^8^, the present study provides a rationale for examining whether HCA could be used to treat other hyperproliferative skin diseases, such as nonmelanoma skin cancers. In addition, the doses used in the study (10 and 30 mg/kg) were lower than those used in anticancer studies (50 mg/kg)^20,21,24^. Since no obvious signs of tissue damage were evident in either of these disease models, this study confirms both the effectiveness and safety of using HCA as a treatment for psoriasis.

In summary, our findings suggest that intraperitoneal administration of HCA ameliorates IMQ-induced psoriasis-like symptoms in mice by reducing Th17 immune responses, specifically by inhibiting PKM2-STAT3 signaling in Th17 cells and keratinocytes. Given the importance of STAT3 in psoriasis, HCA-mediated targeting of the PKM2-STAT3 axis represents an effective strategy for treating this disease, as well as other Th17 cell-mediated inflammatory diseases.

## Supplementary information

Supplementary material
