# Supplementary material for: 2′-Hydroxycinnamaldehyde ameliorates imiquimod-induced psoriasiform inflammation by targeting PKM2-STAT3 signaling in mice
Source: Exp Mol Med. 2021 May 14;53(5):875–84. doi: 10.1038/s12276-021-00620-z (PMC8178393; doi:10.1038/s12276-021-00620-z)
Supplement: Supplementary file 1 — Supplementary material [file 12276_2021_620_MOESM1_ESM.pdf]

2′-Hydroxycinnamaldehyde ameliorates imiquimod-induced psoriasiform inflammation via targeting of PKM2-STAT3 signaling in mice

Lihua Hao[1,*], Yuancheng Mao[1,*], Jin Park[2], Byoung-Mog Kwon[3], Eun Ju Bae[4,$] and Byung-Hyun Park[1,$]

[1] Department of Biochemistry and Molecular Biology, Chonbuk National University Medical School, Jeonju, Jeonbuk 54896, Republic of Korea

[2] Department of Dermatology, Chonbuk National University Medical School, Jeonju, Jeonbuk 54896, Republic of Korea

[3] Laboratory of Chemical Biology and Genomics, Korea Research Institute of Bioscience and Biotechnology, Daejeon 34141, Republic of Korea

[4] College of Pharmacy, Chonbuk National University, Jeonju, Jeonbuk 54896, Republic of Korea

Contents

1. Supplementary Figures
2. Supplementary Table

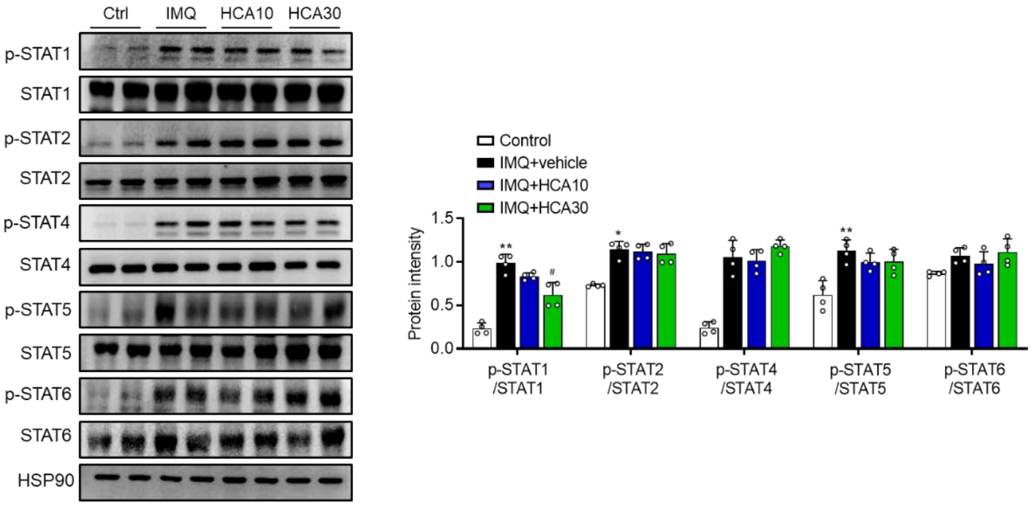

Figure S1. Expression of STAT family members by HCA treatment. All experimental procedures proceeded as described Figure 1's legend. Protein levels of STAT family members in back skin were examined by Western blot. Values are mean ± SD. * $p < 0.05$ and ** $p < 0.01$ versus control; $^{\#}$ $p < 0.05$ versus IMQ+vehicle.

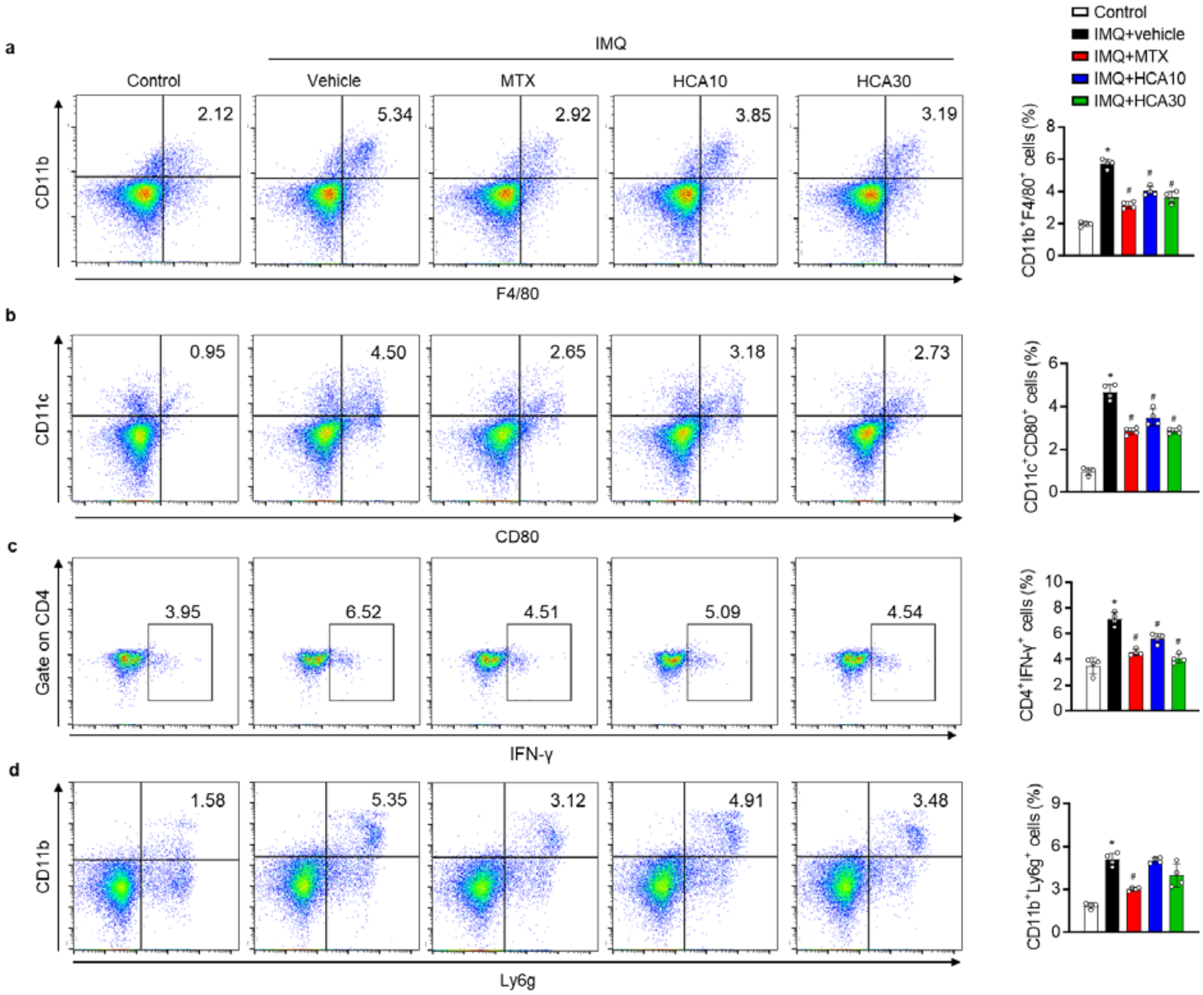

Figure S2. Flow cytometric analysis of back skin tissues. Cell suspensions prepared by enzymatic digestion and gentle dissociation of back skin tissue were analyzed by flow cytometry. Representative flow plots and subpopulations of macrophages (**a**, $CD11b^{+}F4/80^{+}$), dendritic cells (**b**, $CD11c^{+}CD80^{+}$), Th1 cells (**c**, $CD4^{+}IFN\text{-}\gamma^{+}$), and neutrophils (**d**, $CD11b^{+}Ly6g^{+}$) are shown. Values are mean ± SD. * p < 0.05 versus control; $^{\#}$ p < 0.05 versus IMQ+vehicle. HCA10, HCA 10 mg/kg; HCA30, HCA 30 mg/kg.

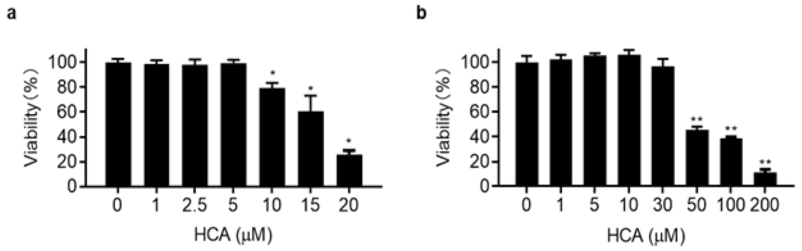

Figure S3. MTT assay of splenic CD4$^+$ T cells and HaCaT cells. Splenic CD4$^+$ T cells (**a**) or HaCaT cells (**b**) were treated with indicated concentrations of HCA for 24 h. Relative cell viability was assessed by MTT assay. Values are mean ± SD (n=4). * $p < 0.05$ and ** $p < 0.01$ versus vehicle.

Table S1. Sequences and accession numbers for primers (forward, FOR; reverse, REV) used in qPCR

Gene	Sequences for primers	Accession No.
Il1b	FOR: GGTCAAAGGTTTGGAAGCAG<br>REV: TGTGAAATGCCACCTTTTGA	NM_008361.4
Il6	FOR: CCACGGCCTTCCCTACTTC<br>REV: TTGGGAGTGGTATCCTCTGTGA	NM_031168.2
Tnfa	FOR: AGGGTCTGGGCCATAGAACT<br>REV: CCACCACGCTCTTCTGTCTAC	NM_013693.3
Il17a	FOR: TCAGCGTGTCCAAACACTGAG<br>REV: CGCCAAGGGAGTTAAAGACTT	NM_010552.3
Ifng	FOR: CTCTTCTTGGATATCTGGAGGA<br>REV: GACTTCAAAGAGTCTGAGGTAG	NM_008377.4
Il22	FOR: GATGAGAGAGCGCTGCTACCTGG<br>REV: GAAGGACGCCACCTCCTGCATGT	NM_016971.2
Il23a	FOR: GCTCCCCTTTGAAGATGTCA<br>REV: GACCCACAAGGACTCAAGGA	NM_031252.2
Rorc	FOR: TGAGGCCATTCAGTATGTGG<br>REV: CTTCCATTGCTCCTGCTTTC	NM_011281.3
Il17f	FOR: TGCTACTGTTGATGTTGGGAC<br>REV: CAGAAATGCCCTGGTTTTGGT	NM_145856.2
Il10	FOR: CTGGACAACATACTGCTAACC<br>REV: GGGCATCACTTCTACCAGGTAA	NM_010548.2
Rora	FOR: GAACACCTTGCCCAGAACAT<br>REV: AGCTGCCACATCACCTCTCT	NM_013646.2
Irf4	FOR: GCAGCTCACTTTGGATGACA<br>REV: CCAAACGTCACAGGACATTG	NM_013674.2
Runx1	FOR: TACCTGGGATCCATCACCTC<br>REV: GACGGCAGAGTAGGGAACTG	NM_009821.3
Ahr	FOR: AGGTGCCTGCTGGATAATTC<br>REV: CCGTCCTTCCCTTTCTTGTT	NM_013464.4
Batf	FOR: CCAGAAGAGCCGACAGAGAC<br>REV: GAGCTGCGTTCTGTTTCTCC	NM_016767.2
Nfkbiz	FOR: GCAGGTAGAGCAGGAAGAAA<br>REV: CCTTGGGCAACAGCAATATG	NM_030612.3
Gapdh	FOR: CGTCCCGTAGACAAAATGGT<br>REV: TTGATGGCAACAATCTCCAC	NM_008084.3
